# Nutrition, IBD and Gut Microbiota: A Review

**DOI:** 10.3390/nu12040944

**Published:** 2020-03-29

**Authors:** Maria Chiara Mentella, Franco Scaldaferri, Marco Pizzoferrato, Antonio Gasbarrini, Giacinto Abele Donato Miggiano

**Affiliations:** 1 UOC di Nutrizione Clinica, Fondazione Policlinico Universitario A. Gemelli IRCCS, Università Cattolica del Sacro Cuore, 00168 Rome, Italy; giacintoabele.miggiano@unicatt.it; 2 UOC di Medicina Interna e Gastroenterologia, Fondazione Policlinico Universitario A. Gemelli IRCCS, Università Cattolica del Sacro Cuore, 00168 Rome, Italy; franco.scaldaferri@policlinicogemelli.it (F.S.); marco.pizzoferrato@hotmail.it (M.P.); antonio.gasbarrini@unicatt.it (A.G.)

**Keywords:** inflammatory bowel disease, nutrition, diet, gut microbiota, microbiome

## Abstract

Inflammatory bowel disease (IBD) is a chronic relapsing–remitting systemic disease of the gastrointestinal tract, characterized by an inflammatory process that requires lifelong treatment. The underlying causes of IBD are still unclear, as this heterogeneous disorder results from a complex interplay between genetic variability, the host immune system and environmental factors. The current knowledge recognizes diet as a risk factor for the development of IBD and attributes a substantial pathogenic role to the intestinal dysbiosis inducing an aberrant mucosal immune response in genetically predisposed individuals. This review focused on the clinical evidence available that considers the impact of some nutrients on IBD onset and the role of different diets in the management of IBD and their effects on the gut microbiota composition. The effects of the Specific Carbohydrate Diet, low fermentable oligosaccharides, disaccharides, monosaccharides and polyols (FODMAP) diet, gluten free diet, anti-inflammatory diet and Mediterranean diet are investigated with regard to their impact on microbiota and on the evolution of the disease. At present, no clear indications toward a specific diet are available but the assessment of dysbiosis prior to the recommendation of a specific diet should become a standard clinical approach in order to achieve a personalized therapy.

## 1. Inflammatory Bowel Disease

Inflammatory bowel disease (IBD) is a heterogenous set of inflammatory diseases, mediated by the immune system, which affect the gastrointestinal tract.

The two main IBD manifestations are Crohn’s Disease (CD) and ulcerative colitis (UC). CD may affect any area of the gastrointestinal tract and its involvement is transmural; colonoscopy findings include skip lesions, cobblestoning, ulcerations and strictures. UC generally occurs only in the colon and involves the mucosa and submucosa only; classically described colonoscopy findings are pseudopolyps and continuous areas of inflammation [1,2].

It is estimated that approximately 3 million people (1.3%) in the US population suffers from IBD [3]. A similar estimate has been provided for Europe [4] with annual direct and indirect costs in the range of billions USD [3,4].

In North America incidence rates for CD range from 0 to 20.2 per 100,000 persons/years and from 0 to 19.2 per 100,000 persons/years for UC. Prevalence ranges from 25.9 to 318.5 cases per 100,000 persons for CD and from 37.5 to 248.6 cases per 100,000 persons for UC. Similarly, in Europe, incidence rates for UC range from 0.9 to 24.0 per 100,000 persons/years and from 0.0 to 11.5 per 100,000 persons/years for CD. Prevalence of UC varies from 2.4 to 294 cases per 100,000 persons, whereas the prevalence of CD ranges from 1.5 to 213 cases per 100,000 persons [5]. In Asia, South America and southern and eastern Europe, the prevalence of IBD is, on average, lower [5,6].

Both incidence and prevalence of IBD are increasing worldwide, and especially in regions usually displaying lower rates, such as Asia and South America [4,5]; furthermore, people migrating to countries displaying high IBD prevalence have a propensity to develop IBD [7] and this is especially true for their first-generation offspring [8].

The etiology of IBD is still not completely understood. Yet, several studies support the hypothesis that its onset is due to a combination and interplay of genetic factors, immune dysregulation and environmental triggers [9,10]. Genetic analysis, which will be discussed in detail in a dedicated section, has led to the discovery of over 230 genes predisposing to IBD [11]. Meaningfully, most of these IBD susceptibility genetic polymorphisms are associated with host mucosal barrier function and are involved in host–microbiome interactions [12,13,14,15,16,17]. These findings support the hypothesis that alterations of the gut microbiome are essential in triggering chronic inflammation and not merely a consequence [18,19]. Further evidence supporting the pivotal role of gut microbiome in the onset of IBD [18] is that CD and UC patients often present a characteristic dysbiosis [20,21,22,23,24,25]; fecal microbiota transplantation seems to induce remission in active UC [26]; the use of antibiotics and probiotics induce and maintain the remission of IBD [27,28,29]; depletion of commensal microbes can result in impaired mucosal healing, chronic mucosal inflammation and colitis [30].

The current theory concerning IBD pathogenesis is that a chronic intestinal inflammation is consequent to an aberrant mucosal immune response that affects genetically predisposed individuals, whose intestinal microbiome undergoes pathologic alterations [18]. Environmental factors, in fact, appear to be pivotal in triggering the onset of the disease given a genetical background predisposing the subject to developing the disease; such a conclusion stems from the observation that the concordance of IBD among monozygotic twins <50% as well as the fact that penetrance of IBD-predisposing gene variants in the general population is incomplete [31,32,33,34]. The role of environmental factors has also been inferred by the analysis of trends in epidemiologic data. The greater incidence and prevalence of IBD in North America and US, as well as the increased risk of IBD for people who emigrate in such regions, and that of their offspring, support a correlation between incidence of IBD and living according to a “Western” lifestyle [9,35]. Main environmental factors, beyond geographical location, that modulate the onset of IBD appear to be diet, smoking, alcohol and drugs (such as nonsteroidal anti-inflammatory drugs and oral contraceptives) [36]. Smoke, alcohol and drugs are supposed to contribute to IBD onset because they may both alter the intestinal epithelial barrier properties [7,9] and have an influence on the microbiota composition [37,38]. Yet, mechanisms underlying such correlation are still not well understood—possibly involving, concerning smoke, molecular pathways for oxidative stress induction and hypoxia, alterations in the composition of mucin and intestinal tight junctions, and changes in acid-base balance [37,39] and, concerning alcohol, mainly its effect on the immune system [38,40], and possibly on gut microbiome [41] even if epidemiological studies on alcohol as a risk factor for IBD have sometimes failed to find a significant correlation [38,42].

Based on the above considerations, the purpose of this review is to give further insights on the relationships between nutrition, microbiome and inflammatory bowel diseases. In particular, we focused on various nutritional approaches, specific food components and microbiome to identify a possible link between them that could influence the evolution of IBD, with the aim of determining a personalized diet for patients affected by UC or CD. To this end, the present investigation takes into account what has emerged from the last 10 years of literature focused on nutritional approaches, microbiome and IBD.

## 2. IBD, Genetics and Epigenetics

The genetic component has a strong influence in the susceptibility of IBD, as studies demonstrated that up to 12% of IBD patients have a family history of IBD [11,43,44]. Until now, genome-wide association studies (GWAS) have identified more than 230 single nucleotide polymorphisms (SNPs) associated with IBD [11,45,46,47]. Among the chromosomes, 110 loci related to the development of IBD have been specifically associated with CD and UC, meaning that the diseases share the same mechanistic pathways such as those involved in the innate immunity (NOD2, IRGM and IL-23 pathway) [48]. The genetic risk locus having the strongest association with IBD is NOD2, which in particular, on chromosome 16 is associated with CD [49,50]. NOD2 codes for a pattern recognition receptor that is pivotal in the host-microbe immune response. NOD2, a cytosol protein, is expressed in monocytes, macrophages, gut epithelial cells (including Paneth cells), and lamina propria lymphocytes, including T cells [51,52,53,54]; it binds the muramyl dipeptide (MDP), a portion of a bacterial cell wall peptidoglycan. Upon binding, assembly of NOD2 oligomer induces activation of NF-κB and MAPK and hence transcription of inflammatory cytokines [55,56]. Impaired NOD2 response to microbiome changes may favor changes in the homeostasis between the host immune system and the microbiome, resulting in increased risk of developing IBD [57]. CD patients with NOD2 mutations also display Paneth cells with altered morphology and diminished secretion of α-defensins, antimicrobial peptides also contributing to the homeostasis between the host immune system and the gut microbiome [58]. Mutations of NOD2 may therefore also alter this pathway, finally contributing to establishing both dysbiosis and inflammation [59]. The exact mechanisms by which NOD2 plays a role in IBD onset are still not clearly defined; these observations, yet, strongly suggest that polymorphism at the NOD2 locus modulates host response to the gut microbiome [11]. The risk variant for IBD located in the nucleotide oligomerization domain containing protein 2 gene (NOD2), has the highest odd risk (OR) of 3.1 in CD. Notably, all other SNPs identified have lower ORs. Other genetic variants identified in several genes such as ATG16L1, LRRK2, and IRGM have been associated with increased risk of IBD. These are all linked to autophagy, a process cells activate to clear cytosolic debris and damaged organelles, possibly involved in the response to intracellular pathogens [60,61,62], whose possible link to IBD is yet to be clarified [63,64]. Again, genetic variants may involve an augmented risk of IBD through alteration of the epithelial barrier function, thinning of the mucus layer, and unfolding of protein due to endoplasmic reticulum stress (genes such as MUC19, ITLN1, FUT2, and XBP1) [65,66].

Recent research has also highlighted a role of epigenetic modification—that is, DNA methylation and noncoding RNAs—in the onset and course of IBD [67,68,69,70]. Genetic variants associated with IBD show, as said, incomplete penetrance, and homozygote twins develop IBD in less than 50% of cases [31,32,33,34] leading to the conclusion that environmental factors play a role as risk factors for IBD incidence. Some environmental factors might modulate the risk of developing IBD by epigenetic modifications [69,71]. At present, evidence of epigenetic modifications in patients affected by IBD exists in the differential expression of specific microRNAs (miRNAs) in the colonic mucosa samples of IBD patients compared to control patients [68] and the presence of specific miRNA in the peripheral blood and tissue of IBD patients [72]. miRNAs are also involved in the differential regulation of cytokines following the immune response to bacteria invasion [73]. miRNAs are small noncoding RNA molecules of approximately 22 nucleotides, secreted by microvescicles in a cell-to-cell communication system and are deputed to regulate multiple target genes or signaling pathways. In the last decade, many studies focused on the emerging role of miRNAs in the development of diseases as well as potential biomarkers for diseases [74]. It has been demonstrated that dysregulation of miRNAs in Th17 cells is implicated in IBD, and that, even if the amount of miRNAs does not change between active IBD and remission IBD, the specific miR-16, miR-21 and miR-223 are highly expressed in IBD with active disease compared to patients with IBD in remission [69,74]. Differences in DNA methylation have been described in UC and CD patients; however, results are not robust and consistent enough to establish a causal-link with gene expression in IBD. In an elegant study by Taman et al., some patterns of hypo- or hyper-methylation have been reconducted to the pathogenesis of CD [75]. These data seem to be supported also by the evidence in a large pediatric IBD population, where specific epigenetic variations in the intestinal epithelium might influence the progression of the disease and might gain prognostic value as biomarkers for the disease [76]. Microbiota influences the activation of some genes associated with hypomethylated active regulatory regions, thus inducing the expression of genes associated with colitis and IBD [77].

## 3. IBD and Microbiota

The human gut microbiota is estimated to contain 500–1000 different bacterial species, as well as fungi and viruses [78], with a number of micro-organisms estimated at 1018 CFU/g, ten times greater than that of the cells of the whole human body. The total amount of genes of the microbiota is estimated to be one hundred times greater than that of the human genome [79]. The intestinal microbiome acts symbiotically to produce vitamins, repress expansion of pathologic organisms and facilitate digestion of dietary substrates, all the while being in constant contact with the host immune system, which it modulates [18]. By competing with pathogens for nutrients and by producing bacteriocin, short-chain fatty acids (SCFA), namely butyrate, acetate and propionate, and hydrogen peroxide, the gut microbiota effectively defends the host against bacterial infections [80,81,82,83,84].

The gastrointestinal microbiota shows a gradient in quantity and diversity from the stomach to the colon, with a limited number of species inhabiting the stomach because of its acidic environment, while increasing in number and diversity from the small to the large intestine. The number of species in the gut has been estimated to be between 500 and 1000 [78]; however, the most represented bacterial phyla (90%) consist of four types: *Firmicutes*, *Bacteroidetes*, *Proteobacteria* and *Actinobacteria* [21,78]. The microbiota composition seems to be dictated by the first inoculum the newborn receives during childbirth, with some differences occurring between natural and cesarean delivery, and between subsequent breast- or formula-feeding [11,85]. After cessation of breast feeding, the reduction of immunoglobulin A (IgA) passage from the mother induces changes in the microbiome, for example, the increase of *Firmicutes* and *Bacteriodetes* [86]. During the first one to three years of life, the immune system and gut microbes develop a dependency relationship, leading to establishment of the host–microbiome homeostasis [87,88,89] destined to remain stable unless there is an occurrence of an illness, the use of antibiotics or considerable changes in diet [90,91].

The microbiota benefits from the mutualistic association with the human body, seeing as though the human intestine is a nutrient-rich environment; however, host diet, lifestyle, hygiene or antibiotic consumption induce rapid and constant changes in gut microbiota composition. The microbiome therefore can change rapidly as a result of variation in the composition of the microbiota.

IBD is clearly associated with intestinal dysbiosis. Changes in the microbiome have a pivotal role in determining the onset of the pathology, when the genetic background of the individual makes him/her predisposed and other concomitant environmental factors intervene [18].

Results of studies aimed at characterizing the microbiota of patients suffering from IBD, even sometimes with checkered results, indicate a generalized decrease in biodiversity, measured by an appropriate parameter—alpha [18]—as well as a reduction in specific taxa including *Firmicutes* and *Bacteroidetes*, *Lactobacillus* and *Eubacterium* [20,21,22,23,24,25]. IBD patients also present a reduction in species producing butyrate [92], a short chain fatty acid positively modulating intestinal homeostasis [93,94] and reducing inflammation [95].

A concomitant taxonomic shift, with a relative increase in *Enterobacteriaceae*, including *Escherichia coli* and *Fusobacterium* has also been observed [96]. Joossens et al. (2011) observed in CD patients increased *Ruminococcus gnavus* and decreased *Bifidobacterium adolescentis, Dialister invisus, Faecalibacterium prausnitzii*, alongside an unspecified member of *Clostridium cluster XIVa* [97]. Overall, there is a consensus for a reduction in the total number of species and a decrease in diversity of the microbiota in IBD.

In an elegant study by Lloyd-Price et al. (2019) [98], 132 IBD patients were recruited to identity their molecular profiles and to evaluate microbial activity during the course of the disease. Authors observed a functional dysbiosis in the gut microbiome during flairs of the disease with impaired microbial transcription and, concerning the composition of microbiota, facultative anaerobes were increased at the expense of obligate anaerobes.

## 4. Nutrients

The following paragraphs will address the impact of fats, proteins, carbohydrates and fibers on the onset of IBD and how they can influence the progression of the disease. As far as we know, the incidence of IBD is raised when the Western diet becomes popular, in particular in those countries where it was previously at low-incidence, such as southern Europe and Asia, resulting in the speculation that the nutritional approach might be correlated to the development of the disease [99].

### 4.1. Fats

The casual relationship between a high fat intake diet (HFD) and IBD was first hypothesized when an increase in incidence of CD was observed following the introduction of margarine in Europe at the beginning of the 20th century [100] and later in studies on the Japanese population, correlating fat consumption and incidence of CD and UC [101,102]. This association is now well-established, on the basis of different case-control diet studies and an HFD is regarded as a certain risk factor for developing IBD. More in-depth studies highlight a different impact on disease pathogenesis of different types of fats; particular attention has been paid to the different role of ω-3 and ω-6 polyunsaturated essential fatty acids (PUFA) with several studies demonstrating that ω-3 PUFA is anti-inflammatory, whereas ω-6 PUFA is pro-inflammatory and a balanced ratio of ω-3 to ω-6 PUFA is essential for homeostasis [103]. Indeed, Western diets usually involve a high ω-6 to ω-3 ratio, leading to a greater probability of developing IBD [101,104].

Other fats involved in increasing the risk of developing IBD are long-chain triglycerides (LCT), that prompt intestinal lymphocyte proliferation and up-regulate pro-inflammatory mediators [105]. Medium chain triglycerides (MCT), instead, suppress production of interleukin-8 (IL-8)—a neutrophil attractant mediator overexpressed in the mucosa of IBD patients [106,107]—and are therefore anti-inflammatory [9].

Increased risk consequent to HFD diet may be due both to increased intestinal permeability and to the alteration of the intestinal microbiota. Indeed, most healthy subjects following an HFD diet for one month had their plasma endotoxins levels increase, even if they did not develop inflammation [108]. The mechanism underlying increased permeability may involve under-expression of occludins, some proteins forming epithelial tight junctions [9]. Animal studies clearly show that the HFD diet alters the microbiota, favoring pathobiont expansion [109,110,111], similar to those observed in IBD patients [112].

### 4.2. Proteins

Recent studies connect high protein intake with changes in IBD incidence, suggesting high protein intake from different sources, including red meat, fish, eggs, milk, cheese, nuts may be also a factor modulating IBD incidence [9]. A prospective two-year survey of 67.581 middle-aged women showed that animal protein from fish or meat, excluding those from eggs or dairy, was correlated to increased IBD development [113]. An additional prospective study on the clinical course and relapse of UC patients showed that high meat intake was associated with a significantly increased risk of relapse [114]. Yet, other studies, on a large number of patients, failed to find an association between high protein intake and increased UC incidence [115]. Mechanisms underlying the role of proteins as a factor modulating the onset of IBD remain largely unknown [9]. It has been speculated that animal protein degradation in the gut may produce substrates favoring the expansion of pathobionts, or SCFAs modulating the function of enterocytes [95,114].

In particular, some metabolites coming from protein fermentation, such as ammonia and total sulfide, seems to be increased in UC patients when compared to healthy subjects [116]. As biological consequences, the mucus layer undergoes remodeling in terms of loss of cell and mucus increasing paracellular permeability. Other metabolites, deriving from normal protein degradation, can be considered harmful in such conditions of altered microbiota and gut inflammation:

1. phenolic compounds, the products of aromatic amino acids fermentation by *Bacteriodetes* spp and some *Firmicutes* (phenylacetic acid, phenols, indoles and *p*-cresol), have an in vitro damaging effect on the mucosal barrier function that depend, in vivo, on the presence of other nutrients
2. *N*-nitroso compounds have carcinogenic potential via DNA alkylation
3. polyamines (putrescine, spermidine and spermine) might affect the expression of a particular cotransporter for monocarboxylates such as lactate, pyruvate, leucine and many others, which contribute to the regulation of central metabolic pathways and insulin secretion
4. the metabolism of nitric oxide (NO), deriving from arginine, produces prooxidant species in IBD
5. unabsorbed bile acids influence the balance between acid sensitive/tolerant bacteria shifting toward the latter [116].

Noteworthy, polyamines are also exploited by several pathogens such as *Shigella flexneri*, *Streptococcus pneumoniae*, *Salmonella enterica*, *Helicobacter pylori* to increase their virulence [117].

When considering protein fermentation, of particular relevance are the effects on the matrix produced by the mucus barrier. The mucosal matrix plays a fundamental protective role in the gut, balancing the microbiota and preventing harmful bacteria from contacting the intestinal epithelium. In UC patients the mucus layer is thinner than in healthy subjects, displaying altered mucin composition, such as altered *O*-glycosylation of MUC2, the main mucin secreted. Mucins of affected subjects have impaired glycosylation, sialylation and sulfation that in remission phases can shift to normal levels [118]. *Ruminococcus torques* is particularly active in mucin degradation. Conversely, CD patients present increased MUC2 expression and reduced sulfation and glycosylation altering mucus viscoelastic properties during acute inflammation.

Taken together, these observations pinpoint IBD mucins alterations, due to increased rates of pathogen colonization and to their metabolism, as strongly impacting the worsening of the disease.

### 4.3. Carbohydrates

Carbohydrates show a different absorption profile within the intestine according to their degree of polymerization [9]. The small intestine hydrolyzes and absorbs simple sugars (glucose, fructose, sucrose and starch) while the microbial species in the large intestine degrade fructooligosaccharides and galactooligosaccharides, together with inulin. Insoluble fibers are not digested and increase the bulk of the feces [9,119].

Early studies in the late 1970s first suggested carbohydrates could be a risk factor for CD [120]; later, several studies highlighted a correlation between high sugar and low fiber intakes with IBD, and especially with CD incidence, with a different effect of different carbohydrates [121,122]. A possible mechanism underlying the effects of carbohydrates on gut microbiota is an imbalance in intestinal absorption leading to differential sugar profiles being available in the intestinal lumen, favoring the overgrowth of specific pathobionts [9]. This hypothesis is consistent with the observation that fructose malabsorption and lactose intolerance are associated with IBD [123] and with observations on animals showing that high carbohydrates intake favors dysbiosis [124]. Such observations have led to the formulation of several low- or selective-carbohydrate intake diets (see following paragraph).

Low fiber intake has also been associated with increased IBD incidence [7,100,104,121,122]. Fibers are fermented within the colon, where they promote bacterial diversity, preserve mucosal barriers and prompt the production of SCFA that, in turn, positively modulate intestinal homeostasis [93,94] and reduce inflammation [95]. As said, IBD patients show a decrease in butyrate producing bacterial species, as well as a decreased expression of butyrate transporters [92,125].

## 5. Dietary Additives

Food additives are used to preserve and enhance food quality and improve the taste of processed foods. They can be coating and coloring substances, fillers or stabilizers. In recent years, attention has been given to the effects of dietary additives in the evolution of inflammatory bowel diseases [126]. It has been speculated that elements, such as carrageenan, are a source of sulfur for sulfate-reducing bacteria (SRB) such as *B. wadsworthia*. The hydrogen sulfide (H_2_S) generated has been shown to have detrimental inflammatory effects in the colon, including DNA damage. Emulsifiers, detergent-like molecules as carboxymethylcellulose and polysorbate-80, are widely present in processed foods. They might induce damage in the mucus layer with ensuing alteration in the microbiome and worsening of colitis in animal experimental models [127,128]. Other agents are maltodextrin, used as filler or thickener (it affects gut microbiota, impairs mucus layer and can be involved in necrotizing enterocolitis), noncaloric artificial sweeteners largely present in many common beverages (induce dysbiosis and mucosal inflammation), inorganic nanoparticles, food colorants such as titanium dioxide (it has been shown to induce intestinal inflammation and to increase oxidative stress in mice) and antimicrobial agents (damage intestinal microvilli and impair intestinal epithelial barrier) [126].

## 6. IBD and Diets

The scenario outlined in the previous paragraphs, underlines how IBD incidence and course depend on the interplay between genetic predisposition and exposure to different environmental factors, including food intake, with certain food components possibly exerting a negative effect, while others possibly exerting a positive one. Given this scenario, an increasing interest has been given to diet as an easily modifiable environmental factor and, therefore, as a possible preventive or treatment option for IBD [99]. Indeed, IBD patients themselves attribute more importance to diet in affecting their symptoms, than to pharmaceutical treatment [129].

### 6.1. The Specific Carbohydrate Diet (SCD)

The Specific Carbohydrate Diet (SCD) was developed in the 1920s as a treatment for the celiac disease and given the positive results of its application in treating UC [130] was later proposed as an approach for managing IBD [131]. SCD, to be followed for one year during active flares and then for one additional year (and later resumed if symptoms reappear), involves excluding more complex carbohydrates, on the basis that when they reach the colon, being still undigested, they cause fermentation and overgrowth of bacteria and yeasts, switching the microbiome toward a pro-inflammatory profile, finally causing IBD [132,133]. Simple (mono-) saccharides are, instead, included. Allowed foods include unprocessed meats, most fresh vegetables and fruits, all fats and oils, aged cheeses and lactose-free yogurt. Prohibited foods include milk, grains, soft cheeses and non-honey sweeteners [99]. When re-switching to an uncontrolled diet, reintroduction of prohibited food occurs one food type at a time.

### 6.2. The Low FODMAP Diet

The low FODMAP diet involves, similar to SCD, a reduction in poorly absorbed and highly fermentable carbohydrates (monosaccharides, disaccharides, oligosaccharides and polyols), with the difference that monosaccharide intake is favored in SCD, while it is discouraged in FODMAP; the premise underlying the two diets is similar, i.e., that carbohydrates that are poorly absorbed may lead to large intestine dysbiosis, inflammation, fermentation, water secretion and lumen distension [134,135,136]. Foods high in FODMAPs that should therefore be excluded in the low FODMAP diet include high-lactose dairy, excess fructose vegetables/fruits and food rich in fructans/galactans and polyols. Low, regulated consumption of foods with moderate FODMAPs is allowed. Low FODMAPs foods such as dairy free from lactose, low fructans and galactans from vegetables and low fructose are allowed. At the beginning of this nutritional approach, patients should follow an initial 4–6 weeks of strict FODMAP diet adherence, followed by subsequent re-introduction of FODMAPs while monitoring symptoms, with the aim of reaching a FODMAP consumption that still manages symptoms [137]. The FODMAP diet should be followed under oversight of a dietitian, to avoid risk of micronutrient deficiencies or, worse, malnutrition [99].

### 6.3. The Gluten-Free Diet

The gluten-free diet has a clear role in managing celiac disease, involving elimination of gliadin. Allowed foods include gluten-free grains from corn and rice, fresh poultry or meat, fruits, vegetables and dairy; this diet has also been practiced by subjects suffering from non-celiac gluten sensitivity (NGCS), that is, individuals showing improvement of IBS-like symptoms when eliminating gluten, even if lacking of the genetic and immunological features defining the celiac disease [99,138,139]. How this diet may benefit IBD patients is less clear [99]. A possible mechanism may involve the inactivation of the immune system by amylase-trypsin inhibitors (proteins found in wheat and commercial gluten) and/or wheat germ agglutinin, as in NGCS [139,140,141], but gliadin might also increase intestinal permeability, translocation of bacteria and immune response interfering with epithelial tight junctions [142]. Further, the gluten-free diet also involves low FODMAPs consumption, with the consequent possible benefits already outlined for that approach [139,140]. Again, the gluten-free diet should be undertaken under supervision of a competent specialist because of its potential implications, including micronutrient and dietary fiber deficiencies [143].

### 6.4. The Anti-Inflammatory Diet

The anti-inflammatory diet is based on the aim of reducing inflammation by intake of anti-inflammatory phytonutrients and spices and omega-3 polyunsaturated fatty acids (from fish). The individual is advised to daily intake fruits and vegetables, providing anti-inflammatory compounds like vitamins B3, B6, E, C, beta-carotene as well as zinc and magnesium. Animal proteins are allowed but plant proteins from legumes are recommended [99]. A practical application has been provided by Olendzki et al. [144] who developed an anti-inflammatory diet for IBD patients, called nutritional regimen for IBD (IBD-AID). This diet differs from SCD as it allows for consuming some grains, gluten and probiotic foods, aimed at addressing some of the deficiencies of SCD and involves taking omega-3 fatty acids while decreasing total and saturated ones. The regimen develops into four phases with different food categories and texture [144].

### 6.5. The Mediterranean Diet

The Mediterranean diet is somewhat similar to the anti-inflammatory one as it involves intaking phytonutrients, unsaturated fats such as olive oil replacing saturated and trans-fatty acids, omega-3 polyunsaturated fats, vegetables, high-fiber whole grains, nuts and low intake of red meats [145]. Adherence to this diet has been correlated to a decrease in inflammatory markers [146,147]. This diet appears promising as a possible strategy to tackle IBD as evidence exists [104], when considering pre-illness diets, that high fruit and fiber diets protect against CD, and a great vegetable intake prevents the develop of UC, while high intake of meats, omega-6 fatty acids, polyunsaturated fatty acids, and total fats are associated with increased incidence of CD and UC. Different to other dietary approaches, the Mediterranean diet is less prone to expose the patient to nutritional deficiencies [99,145].

### 6.6. Other Nutritional Interventions

Other nutritional interventions rely on observations of different IBD incidence rate according to exposition to different food components. For example, given the anti-inflammatory effect of ω-3 PUFA outlined in Section 4, ω-3 PUFA have been investigated as supplementing agents possibly allowing to manage IBD; indeed, several reports showed that supplementation of ω-3 PUFA decreases inflammatory parameters but has no effect on disease activity or relapse rates [148,149,150,151]. Furthermore, two clinical trials on the supplementation with ω-3 PUFA showed mixed results for UC and concluded that supplementation cannot prevent CD relapse [152,153]. Instead of mere supplementation, results of another investigation indicate that a different approach, by balancing ω-3 and ω-6 PUFA, could be more effective, as IBD patients with a unitary ω-3/ω-6 ratio showed a higher remission rate [154,155]. Similarly, the observation that long chain triglycerides (LCT) rich diets increase IBD incidence, suggests that low-LCT diets might be effective in inducing IBD remission [156,157]. However, more clinical evidence is needed to determine if fats with anti-inflammatory effects may be therapeutically advantageous in IBD [9].

## 7. Diets Effectiveness and Impact on Microbiota

Considering the mechanisms of action underlying how the different diets outlined in the section might be effective in managing or even treating IBD, two main—and interconnected—action modalities emerge: a possible direct modulation of inflammation or immune response and a positive effect on the microbiota/microbiome, with the latter based on knowledge about gut microbiota composition and metabolism, on the degradation pathways of different food components and on the observation that diet seems to have a crucial influence on microbiota composition and function both when switching from a vegetarian to an animal-food based diet [158,159] and from a high-fat/low fiber to a low-fat/high fiber diet [160].

### 7.1. The Specific Carbohydrate Diet (SCD)

In a survey of 50 subjects affected by IBD and self-treating with the SCD, the clinical remission was observed in 66% of patients after about 10 months following the nutritional regimen. Furthermore, numerous subjects were able to discontinue corticosteroid therapy [161]. The authors specifically indicate changes in intestinal microbiome they previously observed as a contributory mechanism explaining the positive results they observed [161]. An anonymous online survey completed by 417 adult patients suffering of IBD (47% CD, 43% UC, and 10% indeterminate colitis) and following the SCD, showed that 33% and 42% of patients experienced symptomatic remission after 2 and 6–12 months of diet, respectively [162]. Among the outcomes self-assessed by patients, abdominal pain was improved as well as improvements regarding diarrhea, blood in the stool, limitations of activities and weight loss. Of note, concerning the impact of SCD on the microbiota and microbiome, twelve pediatric patients aged 10 to 17 with mild to moderate IBD and subjected to SCD diet for 12 weeks underwent significant clinical improvement; the authors observed a distinctive dysbiosis for each individual in most pre-diet microbiomes ending in significant changes in microbiota composition after dietary switch. Interestingly, changes were not consistent in all patients (with contrasting results regarding even microbial diversity, where some patients showed increasing post-diet diversity, others showed a decrease) [163].

A case report of a young lady with a UC diagnosis and assuming SCD, showed that the diet successfully improved all UC symptoms and induced a dramatic variation of the microbiome. Prior to the SCD regimen, the most abundant species were *Fusobacterium ulcerans* and *Viellonella dispar*. In that study, the microbiota of the case subject was compared to that obtained from three healthy subjects with no restriction diet. None of the species *Fusobacterium ulcerans* and *Viellonella dispar* were found in the control subjects, where instead the dominating species were *Bacteriodeaceae, Ruminococcaceae* and *Lachnospiraceae*. After two weeks of diet, the patient’s microbiome showed a decrease of *Fusobacterium ulcerans* alongside a marked increase of many *Enterobacteriaceae* species [164].

In another trial, six subjects with CD compared to two healthy controls, were treated with SCD or low residue diet for thirty days. Fecal samples were evaluated at day 1 and day 30. At baseline, the results, consistent with previous findings, showed a reduced microbial diversity in CD patients. The most increased classes were *Clostridia* and *Gammaproteobacteria* and some species of the *Phylum Bacteriodetes*, while *Clostridium lactifermentans* was reduced. After the SCD regimen, the microbial diversity increased with a high prevalence of nonpathogenic species of the clostridia family. However, no clinical significant improvement was observed [165].

On the whole, robust data—especially on adults—are lacking, and prospective investigations, possibly through comparative case-control studies, are warranted to get an in-depth understanding of how the SCD may impact the microbiota and the microbiome [99].

### 7.2. The Low FODMAP Diet

Concerning the low FODMAP diet, meaningful results have been achieved when using it as a tool to manage symptoms of irritable bowel symptoms (IBS) [166]; such findings are interesting considering that more than 30% of IBD patients also suffer from concomitant IBS [167,168]. When 89 adult IBD patients (28 CD, 61 UC) in clinical remission or with mild-to-moderate disease were randomized to undergo for 6 weeks either a normal diet or a low FODMAP, a significant improvement was observed in terms of quality of life and in terms of reduction of IBS-like symptoms [169]. These findings have been recently corroborated by Bodini et al, who observed an amelioration of the disease alongside a quality of life increase in 26 IBD subjects undergoing a low FODMAP diet compared to IBD subjects under a standard diet in a 6-week period [170].

Serial FODMAP challenges in a randomized, double-blind, placebo-controlled, crossover, re-challenge trial concerning IBS-like symptomatic IBD patients in remission showed that, contrary to galacto-oligosaccharides and sorbitol, the intake of fructans worsened gastrointestinal symptoms in patients with IBD compared to placebo [171]. Low FODMAPs diet in a small randomized, controlled, crossover trial on quiescent CD patients showed improvement in overall gastrointestinal symptoms; 9 patients were randomized to 21 days of low or high FODMAP diets with ≥21-day washout in between. Five-day fecal samples were collected at the end of each diet and analyzed for calprotectin, pH, SCFA and bacterial abundance and symptoms were recorded daily. SCFA, pH and total bacterial abundance remained unaltered; the relative abundance was higher for butyrate-producing *Clostridium cluster XIVa* and mucus-associated *Akkermansia muciniphila* and was lower for *Ruminococcus torques* during the high compared with low FODMAP diet. No effects were observed in calprotectin but the severity of the symptoms was worsened with the high FODMAP diet [172].

Yet, while it is currently accepted that IBD patients may be treated for their IBS-like symptoms according to a low FODMAP approach, little is known concerning how this diet may impact the underlying inflammation [99].

### 7.3. The Gluten-Free Diet

Herfarth and colleagues studied the prevalence of a gluten-free diet and the improvement of clinical symptoms in patients with IBD [173]. They considered 1.647 patients in a cross-sectional study, finding that 0.6% of them had a co-diagnosis of celiac disease and 4.9% reported NCGS. About 20% of the subjects had previously adhered to a gluten-free diet and 8.2% were currently following it; about 66% of patients who had followed the gluten-free diet reported improvement of intestinal symptoms and about 38% reported less severe and frequent IBD flares. Yet another large prospective study involving 1254 patients, most not being diagnosed with celiac disease, found no significant differences between patients adhering to a gluten-free diet and those who did not, concerning disease activity, hospitalization, surgery, or complication [174]. This study found a variation in the microbiota of patients adhering to the gluten-free diet; in fact, alpha diversity analysis tended to be higher in those affected by CD and following a gluten-free diet than in CD patients following a regular diet, with the lowest species richness observed in patients eating meat >4 days per week; in UC patients, the gluten-free group tended to have the lowest species richness, with a trend for the highest species richness in patients eating meat >4 days per week. The authors also observed significant differences in the operational taxonomic units (OTUs) between diet types in CD patients, where several representatives of the phyla *Bacteroidetes* and *Firmicutes* were significantly correlated with regular diet patients when compared to the those adhering to the gluten-free one; they did not observe similar differences in UC ones [174].

### 7.4. The Anti-Inflammatory Diet (AID)

Olendzki et al., who developed the IBD-AID diet, published a study concerning 11 IBD patients who were refractive to pharmacological treatment or had not adequately controlled symptoms [144]. All 11 patients reported an improvement in symptoms and could reduce medications. At present, no other data, either concerning inflammatory markers or microbiome modifications, are available concerning this diet [99].

### 7.5. The Mediterranean Diet

Results from clinical and translational research on the Mediterranean diet point to its possible meaningful use in managing IBD, and thus additional studies could have the potential to add further insights to the field [99]. Concerning published data, it was observed that 153 Italian healthy subjects were investigated for their dietary habits and their gut microbiota was assessed, and high-level adherence to a Mediterranean diet was found to beneficially impact the gut microbiota and associated metabolome [175]. These studies provided the first concrete evidence for the interconnection between Mediterranean dietary patterns, gut microbiota and microbial metabolites as they observed that the consumption of fruit, vegetables and legumes by subjects with satisfactory adherence to the Mediterranean diet was associated with an increase in fecal SCFA levels, an effect that was likely boosted by bacteria belonging to both the *Firmicutes* and *Bacteroidetes* capable of degrading carbohydrates not digestible by the host. When eight adult patients suffering from CD followed the Mediterranean diet for 6 weeks, their transcriptome analysis showed a change in expression of more than 3000 genes; changes in the intestinal microbiota, although not significant, showed a trend towards normalization [176] with an increase in the expression of *Bacteroidetes* (17.89% to 18.74%), *Clostridium cluster IV* (19.2% to 21.86%) and *Clostridium cluster XIVa* (26.78% to 28.79%) and a decrease in the abundance of *Proteobacteria* (5.93% to 5.48%) and *Bacillaceae* (4.65% to 4.21%).

The Mediterranean diet has a high amount of fiber, thus can be unsuitable for patients during flares of the disease but it is highly recommended after remission, with appropriate adjustments. In fact, the use of pulses, containing soluble fibers, has a prebiotic effect promoting the growth of microbial species that produce propionic and butyric acid which decrease inflammatory cytokines expression. Vegetables can be consumed both cooked and uncooked and broccoli especially seems to prevent relapse in CD patients [177]. Fruits can be treated with a juice extractor, to eliminate fibers and the minerals and vitamins contained are involved in the immunomodulation, as well as olive oil and bluefish which have anti-inflammatory effects.

## 8. Conclusions

The individual nutritional status is crucial to one’s overall well-being and is one of the fundamental factors ensuring the appropriate function of the immune system.

By ensuring an adequate intake of nutrients during both flairs and remission phases of the disease, the nutritional approach truly influences the management of IBD.

The evaluation of the last decade’s most relevant literature on the association between nutrition, IBD and microbiome shows that IBD is clearly associated with intestinal dysbiosis and that, at present, no specific nutritional regimen is effective for all CD and UC patients.

The role of many nutrients for developing IBD has been demonstrated; furthermore, several studies have showed that, in IBD patients, specific diets either negatively or positively influence disease symptoms. This effect seems to be associated with a variation in the gut microbiota.

Understanding how to modulate the composition and metabolism of gut microbiota through a nutritional approach could be a strategy to control the disease. The therapeutic goal is to achieve the remission of the disease and, possibly, to maintain an optimal homeostasis and prevent any relapse through a specific and individualized diet.

The hypothesis that nutrition might contribute to achieving and maintaining the remission of the disease is at the same time challenging and attractive. Future perspectives should include investigating the correlation between nutrients and microbiome through appropriate, well-designed and targeted clinical studies.
